# Cofactors, age at onset, allergic comorbidities and gender are different in patients sensitized to omega-5 gliadin and Pru p 3

**DOI:** 10.1038/s41598-022-25368-y

**Published:** 2022-12-02

**Authors:** Giulia Costanzo, Annalisa Matolo, Laura Saderi, Maria Rita Messina, Davide Firinu, Maria Pina Barca, Paolo Serra, Nicoletta Corso, Giovanni Sotgiu, Stefano Del Giacco

**Affiliations:** 1 Department of Medical Sciences and Public Health, University of Cagliari, 09042 Monserrato, CA Italy; 2 Clinical Epidemiology and Medical Statistics Unit, Department of Medical, Surgical and Experimental Sciences, University of Sassari, Sassari, Italy

**Keywords:** Medical research, Respiratory signs and symptoms, Skin manifestations

## Abstract

Aim of this study is to clarify the impact of cofactors on allergic reactions in patients sensitized to LTP and ω-5-gliadin. We retrospectively examined the charts of our outpatients from January 2015 to July 2019 and identified 157 patients seen for urticaria/angioedema or anaphylaxis after a meal, in presence or absence of cofactors and sensitized to LTPs (Pru p 3 and/or Tri a 14) and/or ω-5-gliadin (Tri a 19). we compared LTPs-positive patients and those sensitized to Tri a 19 in order to assess the difference in cofactors involved and in frequency of clinical presentation. Our data show that exercise is the most frequent cofactor in FDEIAn and the involvement of exercise, alcohol and multiple cofactors was more frequently found in males than in females. We found that exercise, pollen counts peaks and multiple cofactors were more often related to anaphylaxis than mild reactions. Finally, we performed a comparison between patients LTPs+ and Tri a 19+ that showed in the latter group a lower frequency of allergic comorbidities, a higher median age at the onset of symptoms and frequency of alcohol exposure. Our data show that the search for possible cofactors involved in food allergy is essential not only for diagnostic purposes, but also for risk assessment strategies.

## Introduction

Food allergy is an adverse reaction following food intake and its severity may range from mild to severe^1,2^. Anaphylaxis is a life-threatening event whose severity depends on type and dose of allergen, and cofactors may play a key role^3,4^. The type of food might vary with age and geography. In the majority of the Mediterranean European Countries nonspecific lipid transfer proteins (LTPs) are considered the main cause of food allergy^5^. LTPs can be usually found on the superficial stratum of fruits, including Rosaceae (e.g., peach, apple, pear, plum, cherry, and apricot), melon, and watermelon, owing to its defensive role^6^.

LTPs hypersensitivity can range from an asymptomatic status to symptomatic condition in sensitized individuals exposed to cofactors^7,8^. The wheat protein ω-5-gliadin (O5G, Tri a 19, a fraction (3–6%) of wheat gluten) is the major allergenic protein in wheat-dependent exercise-induced anaphylaxis (WDEIAn)^9,10^. O5G is quite insoluble and usually poorly absorbed after wheat ingestion; exercise can promote O5G absorption^11^. Concomitant ingestion of alcohol or aspirin can increase plasma gliadin levels^4^.

Cofactors may explain why food ingestion sometimes elicits an anaphylactic reaction and sometimes not^12^. The cofactor more often linked to food reactions is exercise, hence the definition food-dependent exercise-induced anaphylaxis (FDEIAn)^13^, followed by drugs (e.g., NSAIDs)^14^, alcohol, stress, tiredness^15,16^.

Patient history and personal activities can help perform an appropriate diagnosis^17^.

Aim of the present study was to assess the role of cofactors in a cohort of patients sensitized to LTPs and/or to Tri a 19, referred by general practitioners because of urticaria and/or angioedema, or anaphylaxis after food intake.

## Results

A total of 157 patients were recruited [89, 56.7%, were females, median (IQR) age 30 (23–42) years]. The median (IQR) age at the onset of symptoms was 24 (18–37) years.

Overall, 39 (24.8%) patients had a negative history of allergic diseases; 110 (70.1%) suffered from allergic rhino-conjunctivitis, 41 (26.1%) from asthma, 9 (5.7%) from atopic dermatitis, and 31 (19.8%) from a previously diagnosed food allergy.

Twenty-four (15.3%) patients had a history of gastric disease: nineteen (79.1%) patients gastroesophageal reflux disease, one (4.2%) hiatal hernia, one (4.2%) peptic ulcer, and three (12.5%) chronic gastritis.

Ninety (57.3%) patients had a familiarity for allergy.

Anaphylaxis was the most common (52.9%) clinical condition. The most frequent symptoms were angioedema (87.3%), urticaria (82.8%), dyspnea (25.5%), itching (14%), and syncope/pre-syncope (11.5%). The median (IQR) time from food consumption to symptom onset was 60 (30–120) min. Overall, 91/157 (58%) patients experienced symptoms with ≥ 1 cofactor. Exercise was found in 31.9%, humid-heat in 31.2%, pollen peak in 17.8%, NSAIDs in 15.9%, alcohol in 9.6%, and PPI (pump proton inhibitors) in 4.5%. Fifty-two patients reported more than one cofactor, with the most frequent combination including exercise, humid-heat, and pollen peak in 19.2% of the cases, followed by exercise and humid-heat in 17.3%, exercise and NSAIDs in 11.5%.

Exercise was the most prevalent cofactor in males (41.2% vs. 24.7%, *p*-value: 0.03), as well as alcohol (19.1% vs. 2.3%, *p*-value: 0.001) and multiple cofactors (42.7% vs. 25.8%, *p*-value: 0.03). No differences were found for clinical reactions between males and females (Table 1). Exercise, pollen peak, and multiple cofactors were significantly associated with an increased frequency of severe reactions (Table 2).

**Table 1:** Differences between males and females regarding the type of clinical presentation and the type of cofactors involved.

	Male (n = 68)	Female (n = 89)	*p*-value
Mild reactions	29 (42.6)	45 (50.6)	0.33
Anaphylaxis, n (%)	39 (57.4)	44 (49.4)	
Physical activity, n (%)	28 (41.2)	22 (24.7)	0.03
Humid heat, n (%)	23 (33.8)	26 (29.2)	0.54
Pollen peak, n (%)	13 (19.1)	15 (16.9)	0.71
NSAIDs, n (%)	12 (17.7)	13 (14.6)	0.61
PPI, n (%)	4 (5.9)	3 (3.4)	0.47
Alcohol, n (%)	13 (19.1)	2 (2.3)	0.001
> 1 cofactor, n (%)	29 (42.7)	23 (25.8)	0.03

**Table 2:** Relationship between type of clinical presentation and cofactors.

	Mild reactions (n = 74)	Anaphylaxis (n = 83)	*p*-value
Physical activity, n (%)	15 (20.3)	35 (42.2)	0.003
Humid heat, n (%)	22 (29.7)	27 (32.5)	0.71
Pollen peak, n (%)	8 (10.8)	20 (24.1)	0.03
NSAIDs, n (%)	10 (13.5)	15 (18.1)	0.44
PPI, n (%)	4 (5.4)	3 (3.6)	0.71
Alcohol, n (%)	6 (8.1)	9 (10.8)	0.56
> 1 cofactor, n (%)	14 (18.9)	38 (45.8)	< 0.0001

The most frequently suspected foods were wheat (29.3%), peach (23.6%), tomato (22.9%), peanut (20.4%), walnut (19.1%), and hazelnut (17.8%). A difference was found between patients with FDEIAn and those with “classic” food allergy (Table 3).

**Table 3:** Comparison of foods between FDEIAn and “classic” food allergy.

	Cofactors		No cofactors
Wheat	35/91	Peach	28/66
Tomato	21	Peanut	22
Walnut	14	Walnut	16
Hazelnut	12	Hazelnut	16
Peanut	10	Tomato	15
Peach	9	Wheat	11
Others	28	Others	36

Of the 147 previously treated patients 23 (15.7%) underwent treatment with epinephrine, 110 (74.8%) with antihistamines and/or corticosteroids; after our clinical evaluation, 107 (68.2%) patients were prescribed adrenaline autoinjectors, in addition to a personalized plan with behavioral rules, including avoidance of suspected or documented food and cofactors.

### Allergy test results

The median (IQR) serum total IgE concentration was 184 (79.7–397) kU/L and the median (IQR) tryptase concentration was 3.7 (2.8–4.7) µg/ml. Allergy testing was positive for airborne allergens in 101/108 (93.5%) patients and for food allergens in 110/120 (91.7%) patients.

Sensitization to Pru p 3 was found in 92.1% (139/151) of the cases, with a median (IQR) specific IgE concentration of 5.9 (2.3–15.8) KU/L, whereas 65.5% (55/84) had IgE to Tri a 14, with a median (IQR) specific IgE concentration of 0.9 (0.4–5.6) KU/L; 32.9% (51/155) showed IgE positivity both for Pru p 3 and Tri a 14; 21.3% (20/94) were sensitized to Tri a 19, with a median (IQR) specific IgE concentration of 0.3 (0.2–6.8) KU/L.

Of the 91 patients who experienced symptoms with ≥ 1 cofactor 9 (9.9%) were sensitized to Tri a 19 and 82 (90.1%) to LTPs.

A co-sensitization to Pru p 3 and Tri a 14 was associated with a significant greater risk of developing serious reactions (*p*-value: 0.04), in the absence of a correlation between specific IgE positivity and clinical severity (Tables 4 and 5). A direct correlation between the titers of specific IgE and the size of clinical manifestation in term of life-threatening events was not found. (Table 1 supplementary materials). We did not find a significant statistical correlation between the severity of the reaction and the simultaneous presence of IgE positivity for both Pru p 4 and LTPs (Table 4).

**Table 4:** Association between type of clinical presentation and specific IgE positivity.

	Mild reaction	Anaphylaxis	OR (95% CI)	*p*-value
Pru p 3 and Tri a 14 ≥ 0.1, n (%)	18/73 (24.7)	33/82 (40.2)	2.1 (1.0–4.1)	0.04
Pru p 3 ≥ 0.1, n (%)	65/66 (98.5)	74/75 (98.7)	1.1 (0.1–18.6)	0.93
Tri a 14 ≥ 0.1, n (%)	20/28 (71.4)	35/46 (76.1)	1.3 (0.4–3.7)	0.66
LTP and Bet v 2 positivity, n (%)	1/24 (4.2)	4/23 (17.4)	4.8 (0.5–47.1)	0.17
LTP and Pru p 4 positivity, n (%)	1/4 (25.0)	2/10 (20.0)	0.8 (0.1–11.7)	0.84

**Table 5:** Relationship between clinical severity and specific IgE positivity.

	Pru p 3 negative	Pru p 3 ≥ 0.1	*p*-value
Anaphylaxis, n (%)	6/12 (50.0)	74/139 (53.2)	0.83

	Tri a 14 negative	Tri a 14 ≥ 0.1	*p*-value
Anaphylaxis, n (%)	16/29 (55.2)	35/55 (63.6)	0.45

	Tri a 19 negative	Tri a 19 ≥ 0.1	*p*-value
Anaphylaxis, n (%)	42/74 (56.8)	11/20 (55.0)	0.89

SPTs to food allergens were more frequently positive when specific IgE could be detected (Table 6).

**Table 6:** Relationship between diagnostic results in vivo and in vitro.

	Pru p 3 negative	Pru p 3 ≥ 0.1	*p*-value	CCC (95% CI)
SPT: positivity to food allergens, n (%)	4/8 (50.0)	101/107 (94.4)	0.002	0.40 (0.23–0.54)
Peach, n (%)	0/8 (0.0)	47/105 (44.8)	0.02	0.10 (0.02–0.18)

	Tri a 14 negative	Tri a 14 ≥ 0.1	*p*-value	CCC (95% CI)
SPT: positivity to food allergens, n (%)	14/19 (73.7)	39/41 (95.1)	0.03	0.25 (0.05–0.45)
Wheat, n (%)	2/18 (11.1)	23/41 (56.1)	0.001	0.36 (0.15–0.54)

	Tri a 19 negative	Tri a 19 ≥ 0.1	*p*-value	CCC (95% CI)
SPT: positivity to food allergens, n (%)	51/55 (92.7)	12/16 (75.0)	0.07	− 0.09 (− 0.18; 0.003)
Wheat, n (%)	21/54 (38.9)	8/16 (50.0)	0.43	0.08 (− 0.13; 0.30)

### Comparison between groups

Based on the suspected foods and on clinical and laboratory tests, patients were divided in two groups: Clinical-LTPs+ (146/157, 93%), and Clinical-Tria19+ (11/157, 7%). Clinical-LTPs+ patients which had a reaction in the absence of an identifiable cofactor (64/146) were compared with those with a known cofactor and reported an episode of anaphylaxis or urticaria/angioedema (82/146): median age at the onset of symptoms, median time of onset of symptoms, and frequency of individuals exposed to adrenaline during an acute event were significantly different. However, the type of clinical presentation did not show relevant differences (Table 7).

**Table 7:** Comparison between clinical-LTP + without and with cofactors.

	Clinical-LTP+ without cofactors (n = 64)	Clinical-LTP+ with cofactors (n = 82)	*p*-value
Median (IQR) age, years	30.5 (22.0–41.5)	28.5 (22–40)	0.78
Females, n (%)	41 (64.1)	45 (54.9)	0.26
Median (IQR) age at the onset	21 (15–32)	25 (20–38)	0.02
Familiarity, n (%)	37 (57.8)	49 (59.8)	0.81
Other allergies, n (%)	47 (73.4)	66 (80.5)	0.31
Oculorhinitis, n (%)	44 (68.8)	61 (74.4)	0.45
Asthma, n (%)	20 (31.3)	19 (23.2)	0.27
Atopic dermatitis, n (%)	6 (9.4)	3 (3.7)	0.15
Food allergy, n (%)	11 (17.2)	19 (23.2)	0.38
Non-specific gastric symptoms, n (%)	12 (18.8)	10 (12.2)	0.27
Anaphylaxis, n (%)	30 (46.9)	48 (58.5)	0.16
Time-to-onset of reaction > 1 h, n (%)	8 (15.1)	40 (56.3)	< 0.0001
Median (IQR) time-to-onset of reaction, min	45 (30–60)	90 (60–150)	< 0.0001
SPT: positivity to inhalant allergens, n (%)	41 (91.1)	55 (94.8)	0.46
SPT: positivity to food allergens, n (%)	48 (96.0)	57 (91.9)	0.38
Peach, n (%)	19 (38.8)	30 (49.2)	0.28
Wheat, n (%)	15 (31.3)	15 (24.6)	0.44
Peach-Wheat, n (%)	4 (8.2)	6 (9.8)	0.76
Median (IQR) tryptase, µg/ml	3.6 (2.6–4.6)	3.6 (2.8–4.5)	0.70
Median (IQR) total IgE, KU/L	130 (49–363)	196 (103–385)	0.19
Pru p 3 ≥ 0.1, n (%)	63 (98.4)	76 (98.7)	1.00
Pru p 3 ≥ 0.35, n (%)	62 (96.9)	73 (94.8)	0.69
Median (IQR) pru p 3 IgE values	5.5 (2.1–11.2)	6.4 (2.0–18.6)	0.42
Tri a 14 ≥ 0.1, n (%)	22 (75.9)	33 (73.3)	0.81
Tri a 14 ≥ 0.35, n (%)	18 (62.1)	24 (53.3)	0.46
Median (IQR) tri a 14 IgE values	0.6 (0.2–1.6)	0.4 (0.1–1.9)	0.73
Treatment with adrenaline, n (%)	4 (19.1)	17 (21.5)	0.03
Treatment with Anti H1, n (%)	44 (77.2)	67 (84.8)	0.26
Treatment with steroids, n (%)	47 (82.5)	69 (87.3)	0.43
Adrenaline prescription, n (%)	41 (64.1)	57 (69.5)	0.49

Compared to Clinical-LTPs+ patients, those sensitized to Tri a 19 showed a higher median age at the onset of symptoms, a lower frequency of allergic comorbidities, a higher frequency of alcohol exposure and median total IgE serum concentration. On the contrary, no significant differences were found regarding the type of clinical presentation (Table 8). Interestingly, patients sensitized to Tri a 19 with symptoms and with cofactors (55.6%) experienced anaphylaxis which occurred during physical activity in 80% of the cases.

**Table 8:** Comparison between clinical-LTP+ and clinical-Tri a 19+ with cofactors.

	Clinical-LTP+ with cofactors (n = 82)	Clinical-Tri a 19+ with cofactors (n = 9)	*p*-value
Median (IQR) age, years	28.5 (22–40)	43 (39–46)	0.004
Females, n (%)	45 (54.9)	3 (33.3)	0.30
Median (IQR) age at the onset	25 (20–38)	43 (36–45)	0.002
Familiarity, n (%)	49 (59.8)	3 (33.3)	0.17
Other allergies, n (%)	66 (80.5)	3 (33.3)	0.006
Oculorhinitis, n (%)	61 (74.4)	3 (33.3)	0.02
Asthma, n (%)	19 (23.2)	1 (11.1)	0.41
Atopic dermatitis, n (%)	3 (3.7)	0 (0.0)	1.0
Food allergy, n (%)	19 (23.2)	0 (0.0)	0.20
Non-specific gastric symptoms, n (%)	10 (12.2)	2 (22.2)	0.34
Anaphylaxis, n (%)	48 (58.5)	5 (55.6)	0.86
Time-to-onset of reactions > 1 h, n (%)	40 (56.3)	3 (37.5)	0.46
Median (IQR) time-to-onset of reactions, min	90 (60–150)	60 (37.5–135.0)	0.60
Physical activity, n (%)	45 (54.9)	5 (55.6)	1.0
Humid heat, n (%)	47 (57.3)	2 (22.2)	0.08
Pollen peak, n (%)	27 (32.9)	1 (11.1)	0.27
NSAIDs, n (%)	22 (26.8)	3 (33.3)	0.70
PPI, n (%)	6 (7.3)	1 (11.1)	0.53
Alcohol, n (%)	10 (12.2)	5 (55.6)	0.005
> 1 cofactor, n (%)	48 (58.5)	4 (44.4)	0.49
SPT: positivity to inhalant allergens, n (%)	55 (94.8)	4 (100.0)	1.0
SPT: positivity to food allergens, n (%)	57 (91.9)	3 (50.0)	0.02
Peach, n (%)	30 (49.2)	0 (0.0)	0.03
Wheat, n (%)	15 (24.6)	2 (33.3)	0.64
Peach-Wheat, n (%)	6 (9.8)	0 (0.0)	1.0
Median (IQR) tryptase, µg/ml	3.6 (2.8–4.5)	6.7 (3.5–7.9)	0.10
Median (IQR) total IgE, KU/L	196 (103–385)	492 (286–599)	0.03
Treatment with adrenaline, n (%)	17 (21.5)	2 (22.2)	1.0
Treatment with Anti H1, n (%)	67 (84.8)	9 (100.0)	0.35
Treatment with steroids, n (%)	69 (87.3)	8 (88.9)	1.0
Adrenaline prescription, n (%)	57 (69.5)	8 (88.9)	0.44

## Discussion

FDEIAn is a food allergy where clinical symptoms are elicited by one or more cofactors, such as physical exercise, NSAIDs, PPIs, alcohol, heat-humid climate, pollen peak, menses period, infections^18^. The aim of our study was to evaluate how cofactors influenced the severity. We compared positive-LTPs patients and those sensitized to Tri a 19 to assess cofactors and frequency of clinical presentation (anaphylaxis vs. mild reactions). In agreement with the scientific literature^19^, we show that exercise is the most frequent cofactor in FDEIAn. Exercise, alcohol, and multiple cofactors were more frequent in males. Exercise, pollen peak, and multiple cofactors were more often related to anaphylaxis. In agreement with previous studies, we deem that the basis of the reaction could be related to the increase in bioavailability of allergens due to the augmented gastrointestinal permeability and the decreased threshold for activation of mast cells and basophils^20^.

Similarly to the findings of Cardona et al.^14^, we observed that cereals, tomato and nuts were most frequently involved in FDEIAn and a sensitization to LTPs was reported in 90.1% of our patients. Compared to “classical” food allergy, time-to-onset of reactions was longer and age of onset was higher in FDEIAn. Patients with Tri a 19+ showed a lower frequency of allergic comorbidities, a higher median age at the onset of symptoms, and a more elevated alcohol exposure. The importance of cereals in the Mediterranean diet and the common Italian habit of alcohol consumption during meals could explain this association.

As previously reported, it is known that a potential protective role may be exerted by the simultaneous presence of IgE specific for profilin (such as Pru p 1 and Pru p 4) and Pru p 3 in term of severity of reaction^21,22^. In our cohort we did not find a significant difference in terms of clinical presentation in those positive for Pru p 4, similarly to other studies^23^. However, the rates of sensitization to profilins are low in some areas such as Spain or South Italy^22,24^, thus preventing to fully evaluate this aspect that should be further investigated.

Those who were sensitized to more than 5 nsLTP had an increased incidence of food-induced systemic reaction^25^. Component resolved diagnosis (CRD) was determinant to rule out ω-5-gliadin sensitization in WDEIAn because of the lack of sensitivity and specificity of SPTs^26^. Therefore, CRD helped identify predictive parameters of clinical risk: we found a relationship between Pru p 3 and Tri a 14 co-sensitization and the risk of severe allergic reactions, confirming previous literature^27,28^. In our cohort the intake of NSAIDs was not the most frequent cofactor associated to FDEIAn.

Our study has limitations such as its retrospective nature and the diagnosis not fully confirmed by double-blind challenge test with the culprit food and the cofactor. Nevertheless, avoidance of cofactors when consuming culprit foods often drives to the resolution of the reaction, underlying their true relevance as triggering agents.

FDEIAn needs a diagnostic workup, being potentially linked to severe anaphylactic reactions. The diagnosis is challenging, and collection of a detailed history is mandatory.

The search of possible cofactors involved in allergic reactions to foods is essential not only for diagnostic purposes, but also for risk assessment and to provide appropriate advice to patients. We emphasize that cofactors might enhance food allergy and should always be taken into account when assessing food, alcohol, NSAIDs and exercise allergic reactions.

## Materials and methods

### Patient selection

157 outpatients reporting urticaria/angioedema or anaphylaxis after a meal, sensitized to Tri a 19 and/or to LTPs, from January 2015 through July 2019 were retrospectively enrolled.

Anaphylaxis was diagnosed according to the clinical criteria proposed by Sampson et al.^3,29^, described in the consensus document by the PRACTALL study group^30^. In agreement to Dohi et al., urticaria was typical of FDEIAn (wheal diameter > 10–15 mm, unlike cholinergic urticaria, where it is < 5 mm)^31^. If present in the records, collapse should not have been linked to organ dysfunction (heart, lung, brain) and anaphylaxis should not have occurred in response to warm baths, showers, fever, or other conditions increasing body temperature.

The causal relationship with foods was systematically investigated.

The following variables were collected: age at the admission; age at the onset of symptoms; familiarity for allergic diseases; allergic comorbidities (rhinitis, asthma, atopic dermatitis, history of food allergy); history of gastric disorders (e.g., gastroesophageal reflux disease); symptoms and therapy administered during the acute event (adrenaline, antihistamines, and/or corticosteroids); adrenaline prescription after our clinical assessment; any recurrence during follow-up. Ingestion of foods during the 24 h period preceding the episode and triggering cofactors (e.g., physical exercise, NSAIDs, PPIs, alcohol, heat-humid climate, pollen peak, etc.) were carefully evaluated.

### Ethics

Patients were recruited and enrolled in the study protocol at the Teaching Hospital of the Cagliari University. Written informed consent was obtained from all patients and controls in accordance with the ethical standards (institutional and national) of the local human research committee. The study protocol, including informed consent procedures, conforms to the ethical guidelines of the Declaration of Helsinki and was approved by the Ethics Committee of the Cagliari University Hospital on June 28,2018 (#1127).

### In vivo testing

Skin prick test (SPT) was performed using a panel of commercial allergenic extracts (Lofarma S.p.A., Italy). Inhalants included pollen from grasses (Dactylis glomerata, Festuca elatior, Lolium multiflorum, Phleum pratense, Poa pratensis), Artemisia, a Compositae mixture, Parietaria, Plantago, Chenopodium album, Cynodon dactylon, a ragweed mixture, as well as olive tree, birch, cypress, oak, pine, dandelion, willow tree and ash tree, Dermatophagoides Pteronyssinus and Farinae, dog and cat dander, a feathers mixture, Alternaria alternata, Aspergillus fumigatus, and Penicillium mixture. The panel of food allergens, all available as extracts 1:20 w/v, included a fish mixture (eel, cod, salmon, sardine, mackerel, tuna), meats (beef, pork, and chicken), lobster, shrimp, rice, wheat, maize, potato, tomato, peanut, walnut, almond, kiwi, apple, melon, strawberry, peach, cocoa, egg yolk, egg white, cow casein, β-lactoglobulin, α-lactalbumin and any other food extracts chosen in accordance with the reported clinical history. All SPT were performed using sterile stainless-steel standardized lancets (Heinz Herenz Hamburg^®^, Germany or Stallergenes^®^, France). A SPT was considered positive in case of a wheal diameter ≥ 3 mm surrounded by erythema. Positive responses were rated from 1+ to 4+ based on the relation between the reactions induced by the allergen and the histamine response. Positive and negative controls were adopted using histamine at 10 mg/mL and 0.9% saline, respectively. The double-blind food challenge test (DBFCT) was not performed when sensitization to one or more foods was well documented and the clinical history clearly indicated that the food was tolerated if ingested in the absence of cofactors^32^.

### In vitro testing

Blood samples for in vitro tests were collected and sera were stored at − 20 °C. The tryptase assay was carried out at the first clinical evaluation. Serum total IgE levels were assessed by means of the ImmunoCAP system (Phadia, Uppsala, Sweden) with a detection range from 2 to 5000 IU/L and a reference value < 100 KU/L. Specific IgE detection for food allergens was performed using the ImmunoCAP system (CAP; Phadia). We considered a positive result when the value is ≥ 0.10 kU/L^12^.

### Comparison between groups

Based on the molecular sensitization profile, patients were divided into two groups:

- *Group I* sensitized to LTPs (Pru p 3 and/or Tri a 14)
- *Group II* sensitized to ω-5-gliadin (Tri a 19)

Within each group a further subdivision was performed between those with symptoms with or without at least one cofactor, aiming to assess the influence on severity.

A second comparison was made between positive-LTPs patients and those sensitized to Tri a 19 who showed urticaria/angioedema or anaphylaxis, in both cases, in the presence of cofactors, aiming to evaluate whether a difference in frequency of clinical presentation (anaphylaxis vs. mild reactions) and type of cofactor involved existed.

### Statistical analysis

Qualitative and quantitative variables were collected in an electronic database.

Qualitative variables were summarized using absolute and relative frequencies, whereas quantitative variables using means and standard deviations for those with a normal distribution or medians and interquartile ranges for those with non-normal distribution.

Chi-squared or Fisher’s exact test when appropriate were used to compare qualitative variables, whereas t-test or Mann–Whitney U test were used to compare variables with a parametric and non-parametric distribution, respectively.

Logistic regression analysis was carried out to assess the association between specific IgE positivity and type of reaction. The concordance correlation coefficient (CCC) was calculated to define the agreement between SPT positivity and specific IgE positivity.

The statistical analysis was performed with STATA 16 Statistical Software (StatsCorp, TX) and MedCalc Statistical Software version 19.0.7 (MedCalc Software bvba, Ostend, Belgium; https://www.medcalc.org; 2019). Differences were considered statistically significant when *p*-values were < 0.05.

## Supplementary Information

Supplementary Information.

## Data Availability

More data are available on request contacting the corresponding author.

## Abbreviations

LTP: Lipid transfer protein
NSAIDs: Non-steroidal antinflammatory drug
FDEIA: Food-dependent exercise-induced anaphylaxis
WDEIAn: Wheat-dependent exercise-induced anaphylaxis
PPIs: Proton pump inhibitors
SPT: Skin prick test
