# Supplementary material for: Cofactors, age at onset, allergic comorbidities and gender are different in patients sensitized to omega-5 gliadin and Pru p 3
Source: Sci Rep. 2022 Dec 2;12:20868. doi: 10.1038/s41598-022-25368-y (PMC9718743; doi:10.1038/s41598-022-25368-y)
Supplement: Supplementary file 1 — Supplementary Information. [file 41598_2022_25368_MOESM1_ESM.pdf]

Supplementary materials

Supplementary table 1 Association between type of clinical presentation and specific IgE positivity. Results of analysis performed also using a cut-off for specific IgE positivity of >0.35.

	**Mild reaction**	**Anaphylaxis**	**OR (95% CI)**	**p-value**
Pru p 3 and Tri a 14 ≥0.1, n (%)	18/73 (24.7)	33/82 (40.2)	2.1 (1.0-4.1)	0.04
Pru p 3 and Tri a 14 ≥0.35, n (%)	11/73 (15.1)	26/82 (31.7)	2.6 (1.2-5.8)	0.02
Pru p 3 ≥0.1, n (%)	65/66 (98.5)	74/75 (98.7)	1.1 (0.1-18.6)	0.93
Pru p 3 ≥0.35, n (%)	63/66 (95.5)	72/75 (96.0)	1.1 (0.2-5.9)	0.87
Tri a 14 ≥0.1, n (%)	20/28 (71.4)	35/46 (76.1)	1.3 (0.4-3.7)	0.66
Tri a 14 ≥0.35, n (%)	14/28 (50.0)	28/46 (60.9)	1.6 (0.6-4.0)	0.36
LTP and Bet v 2 positivity, n (%)	1/24 (4.2)	4/23 (17.4)	4.8 (0.5-47.1)	0.17
LTP and Pru p 4 positivity, n (%)	1/4 (25.0)	2/10 (20.0)	0.8 (0.1-11.7)	0.84
